# Complement lectin pathway components MBL and MASP-1 promote haemostasis upon vessel injury in a microvascular bleeding model

**DOI:** 10.3389/fimmu.2022.948190

**Published:** 2022-08-12

**Authors:** Murielle Golomingi, Jessie Kohler, Lorenz Jenny, Elaissa T. Hardy, József Dobó, Péter Gál, Gábor Pál, Bence Kiss, Wilbur A. Lam, Verena Schroeder

**Affiliations:** ^1^ Experimental Haemostasis Group, Department for BioMedical Research, DBMR, University of Bern, Bern, Switzerland; ^2^ Wallace H. Coulter Department of Biomedical Engineering, Georgia Institute of Technology and Emory University, Atlanta, GA, United States; ^3^ Institute of Enzymology, Research Centre for Natural Sciences, Budapest, Hungary; ^4^ Department of Biochemistry, Eötvös Loránd University, Budapest, Hungary

**Keywords:** haemostasis, complement system, mannan-binding lectin (MBL), MBL-associated serine protease-1 (MASP-1), microfluidics

## Abstract

**Background:**

Complement lectin pathway components, in particular mannan-binding lectin (MBL) and MBL-associated serine proteases (MASPs) have been shown to interact with coagulation factors and contribute to clot formation. Here we investigated the role of MBL and MASP-1 in the haemostatic response following mechanical vessel injury in a human microfluidic bleeding model.

**Methods:**

We studied haemostasis in a microvascular bleeding model in the presence of human endothelial cells and human whole blood under flow conditions. We monitored incorporation of proteins into the clot with fluorescently labelled antibodies and studied their effects on clot formation, platelet activation, and bleeding time with specific inhibitors. Platelet activation was also studied by flow cytometry.

**Results:**

Upon vessel injury, MBL accumulated at the injury site in a well-defined wall-like structure. MBL showed partial colocalisation with fibrin, and strong colocalisation with von Willebrand factor and (activated) platelets. Flow cytometry ruled out direct binding of MBL to platelets, but confirmed a PAR4- and thrombin-dependent platelet-activating function of MASP-1. Inhibiting MBL during haemostasis reduced platelet activation, while inhibiting MASP-1 reduced platelet activation, fibrin deposition and prolonged bleeding time.

**Conclusion:**

We show in a microvascular human bleeding model that MBL and MASP-1 have important roles in the haemostatic response triggered by mechanical vessel injury: MBL recognises the injury site, while MASP-1 increases fibrin formation, platelet activation and shortens bleeding time. While the complement lectin pathway may be harmful in the context of pathological thrombosis, it appears to be beneficial during the physiological coagulation response by supporting the crucial haemostatic system.

## Introduction

The complement system is an immune mechanism mediating both recognition and elimination of foreign bodies through proteolytic cascade reactions. It is one of the first lines of defence against pathogens and is also responsible for the enhancement of humoral immune responses (1, 2). Haemostasis is the process that prevents and stops bleeding through actions of the vessel wall, blood cells and the coagulation cascade. A common characteristic of complement and coagulation, which share a common evolutionary origin, is that they consist mainly of serine proteinases together with their activators and inhibitors (3, 4). Both systems act as innate defences against external threats since the presence of foreign or altered cellular surfaces is required for initiation of both pathways (5, 6).

Several decades ago, it was observed that higher levels of complement activation products were found in human serum than in anticoagulated blood, suggesting complement activation during blood clotting (7). Since then, numerous publications have confirmed close interactions between complement and coagulation, summarised in several recent review articles (5, 6, 8). For example, complement C5a was suggested to be generated in the absence of C3 due to proteolytic activation of C5 by thrombin (9). Several studies have shown procoagulant effects of complement activation on the coagulation cascade. The initiating components of the lectin pathway, mannan-binding lectin (MBL) and its binding partners MBL-associated serine proteases (MASPs), MASP-1 and MASP-2, play a role in the activation of prothrombin and subsequent generation of fibrin (10–14). MBL, MASP-1 and MASP-2 have been shown to bind to fibrin (15, 16). MBL and MASP-1/-3 knockout mice showed prolonged bleeding times (17). Besides the coagulation cascade, the complement system has also been associated with platelet activation. For example, C3a anaphylatoxin has been shown to activate platelets (18) and induce formation of the C5b-9 complex on platelets (19). There is also evidence that the complement lectin pathway may be involved in thrombosis: In ischaemic stroke, MBL deficiency was associated with a better clinical outcome (20). MBL and MASP-1/-3 knockout mice showed less FeCl3-induced thrombogenesis (21). Although the data published so far represent many pieces of the puzzle, open questions regarding a possible physiological role of the complement system in haemostasis remain.

It is a challenge to study the processes of coagulation and complement activation under experimental conditions that can recapitulate the (patho-) physiological situation. Many of the factors which can influence these systems, such as the dynamics of blood flow and the presence of endothelial and blood cells, are difficult to implement. Therefore, most *in vitro* studies were performed with plasma or purified proteins. For simulation of physiological conditions, animal models are often used. However, animal models do not always reflect the human physiology and come with several technical and ethical disadvantages. To obtain results which are more compatible with the human physiology and thus more relevant, we use a microvascular, endothelialised, whole blood flow model that applies only human cells and allows to observe the haemostatic response upon a mechanical vessel injury in real-time (22).

The aim of this study was to investigate the role of complement lectin pathway components in haemostasis in a bleeding model under flow conditions in the presence of human endothelial and blood cells and plasma proteins. We monitored incorporation of complement MBL and MASP-1 into the clot and their effects on clot formation, platelet activation, and bleeding time, and elucidated the underlying mechanisms.

## Materials and methods

### Preparation of the microfluidic bleeding model device

The bleeding model devices were prepared from polydimethylsiloxane (PDMS) as described earlier (23). Two 25 cm^2^ flasks were used to grow passage five human umbilical vein endothelial cells (HUVECs) (Lonza, Switzerland) confluently (1x10^6^ cells per flask) over four days in CnT Endo cell culture medium (CELLnTec, Switzerland). Before seeding the cells into the devices, the devices were put under vacuum for 5 min and then filled with a 1 mg/ml collagen solution (rat tail collagen I diluted in ddH_2_O; Thermofisher, Switzerland). The devices were incubated at 37°C for 1h. Two connection tubes (ID 0.02” inch, OD 0.06” inch; Saint-Gobain Tygon, France) filled with collagen solution were inserted into the side and valve channels. The main channel was flushed out with ddH_2_O and filled with a DPBS (Dulbecco’s phosphate buffered saline; Merck, Switzerland) and fibronectin solution (0.005%; Merck). The devices were incubated for 90 min at 37°C. The confluent HUVEC cells were trypsinised for 4 min at 37°C and spun down for 8 min at room temperature (RT). The cell pellet was resuspended in 75µl dextran solution (80 µg/ml diluted in cell culture medium) and filtered through a cell strainer (35 µM; Corning, USA). Fifteen microlitres of the cell suspension (approx. 4x10^5^ cells) were injected into the main channel of each device. The seeded devices were incubated for an hour before being connected to a 10 ml syringe containing cell culture medium CnT Endo and perfused for 48 h at an initial flow rate of 1 µl/min for 12 h followed by 2 µl/min for the remaining 36 h.

### Bleeding experiments

**Figure 1:**
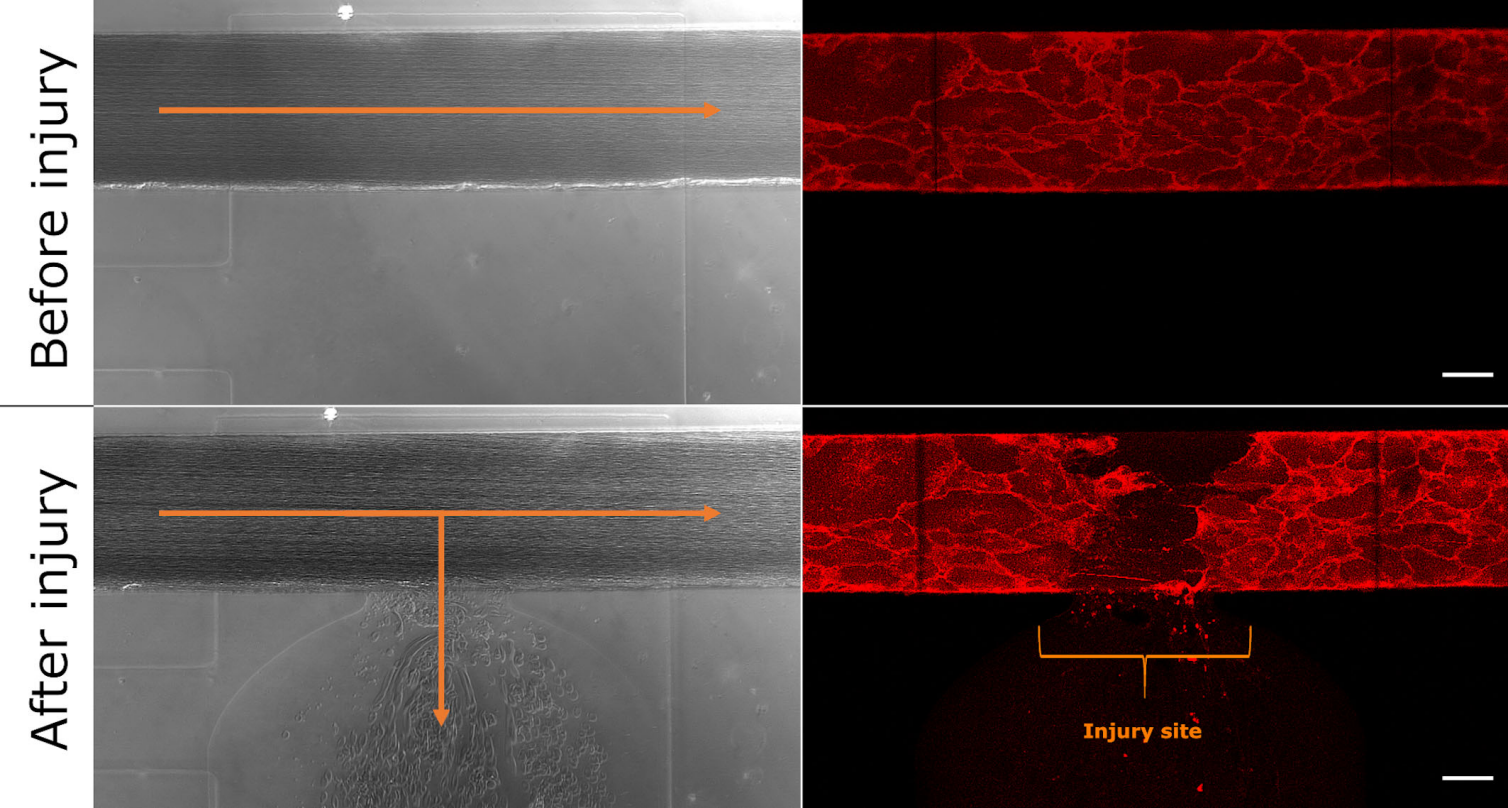
Microfluidic bleeding model before and after injury. From top left to bottom right: Brightfield and CellMask-stained images of the uninjured and injured vessel. The collagen pouch adjacent to the vessel is used to introduce the vessel injury. The blood then flows into the collagen container. Blood flow is indicated with orange arrows. The bracket indicates the injury site. Scale bar: 50 µm.

On the day of the experiment, the confluency of the cells in the main channel was verified. The syringe with cell culture medium was then replaced with a 1 ml syringe containing cell culture medium stained with Cellmask™ Orange plasma membrane stain (1 µl/ml; Thermofisher). The cells were stained for 5 min at a flow rate of 2 µl/min. The device was then installed on the confocal microscope stage (Zeiss LSM 710 with Airyscan). A 10 ml syringe containing cell culture medium was connected to the side channel and a 10 ml syringe containing ddH_2_O was connected to the valve channel. The valve channel syringe was installed on a pump and drawn back to create a 2 ml vacuum. Freshly drawn citrated whole blood from anonymous healthy blood donors (purchased from the blood donation centre Bern, Switzerland) was used for the bleeding experiments. We supplemented 600 µl citrated whole blood with 40 µg/ml corn trypsin inhibitor (CTI; Loxo GmbH, Germany) to prevent contact activation in the syringe and tubing and focus on the extrinsic pathway induced by vessel injury. and added fluorescently labelled antibodies as detailed below. The blood sample was recalcified (final concentration 12.5 mM), drawn up into a 1 ml syringe and used to perfuse the main channel at a flow rate of 2 µl/min. Pressure was exerted ***via*** the side channel syringe to create an injury to the main channel and the recording of images of the injury site (1 image per min for 30 min) was started. **Figure 1** shows the confluent cell monolayer in the device before and after mechanical injury.

### Bleeding time and fluorescence intensity measurements

Each experiment was performed typically 4-6 times, but at least three times, with blood from different blood donors. The bleeding time was determined by eye, meaning that the first frame of the time series, where no blood flow towards the side channel was detected, was defined as bleeding stop. The ImageJ (https://imagej.nih.gov/ij/) software was used to measure the injury size in the first frame. The corrected bleeding time was determined relatively to the control experiment’s bleeding time and injury size. As the maximum running time of the experiment was set to 45 min, if the bleeding did not stop, the bleeding time was recorded as longer than 45 min. For the fluorescence intensity measurements, a region of interest (ROI) at the injury site was selected to measure the different signal intensities. Using the Time Series Analyser plugin of ImageJ (https://imagej.nih.gov/ij/plugins/time-series.html), the signal intensity of the ROI as a function of time was measured. The increase in fluorescent intensity in comparison to the first time point was then calculated.

### Effects of complement lectin pathway components on clot formation

For the localisation experiments, fluorescently-labelled primary or non-labelled primary in combination with fluorescently-labelled secondary antibodies were added to the citrated whole blood: rabbit polyclonal anti-MBL antibody (3µl/600µl of blood; ab189856, Abcam, UK), mouse monoclonal anti-CD41 antibody (5µl/600µl of blood; NB100-2614, Novus Biologicals, USA), mouse monoclonal anti-CD62P antibody clone AK-4 (10µl/600µl of blood; Invitrogen 14-0628-82, Thermofisher), anti-MASP-1 antibody (5µl/600µl of blood; Prestige Antibodies HPA001617, Merck), anti-vWF antibody (3µl/600µl of blood; ab201336, Abcam), AlexaFluor™488-labelled secondary donkey anti-rabbit antibody (1.5µl/600µl of blood; Invitrogen A21206, Thermofisher), AlexaFluor™647-labelled secondary goat anti-mouse antibody (0.75µl/600µl of blood; A21236, Life Technologies, USA). To detect fibrinogen/fibrin, a fibrinogen-AlexaFluor™647-conjugate (10µl/600µl of blood, Invitrogen F35200; Thermofisher) was added. Crosslinked fibrin was detected with the DD-XLink mAb (2µl/600µl of blood; Zedira, Germany).

To assess the functional roles of MBL binding to its targets we measured the bleeding time as well as MBL, MASP-1, CD62P and fibrinogen/fibrin-related fluorescence signal intensities in the presence of the MBL-blocking antibody 3F8 (24) in comparison to those measured in the presence of the non-inhibitory MBL antibody 1C10 (24) (both antibodies were kindly provided by Prof. Peter Garred, University of Copenhagen, Denmark). The concentrations used were 40 μg/ml of 3F8 or 1C10 antibody. The ROI selected was a 300x300 µm^2^ around the injury site.

To assess the functional roles of MASP-1 activity we measured the bleeding time and increase in fluorescence intensity of the CD62P signal in the presence or absence of the MASP-1-specific inhibitor SGMI-1 (S*chistocerca gregaria protease inhibitor* (SGPI)-based MASP-1 inhibitor) (25). The region of colocalisation of CD62P with MBL was selected as ROI.

### Flow cytometry analysis of platelet activation

We used flow cytometry to investigate interactions between complement lectin pathway components and platelets.

To assess if MBL is present on activated platelets, analysis was performed according to the platelet activation protocol of BD Biosciences: 20 µM adenosine diphosphate (ADP) (01905; Merck) was added to 50 µl of whole blood followed after 2 min by fixation with 1 ml of 1% paraformaldehyde (PFA) solution and incubation at 4°C overnight. The cells and platelets were spun down, washed in PBS, and finally resuspended in 1 ml of 2% Fetal Bovine Serum (FBS) solution (diluted in PBS). Fifty microlitres of this suspension were transferred to the wells of a 96-well microplate and the following antibodies were added: an anti-CD41 antibody conjugated with allophycocyanin (APC) (0.25µl/well; Invitrogen, clone HIP8; Thermofisher) was used to identify the entire platelet population, and an anti-CD62P antibody conjugated with BV421 (0.25µl/well; clone AK-4 304926, Biolegend, USA) was used to identify the activated population. To detect MBL, a primary rabbit antibody against MBL (0.5µl/well; ab189856; Abcam) was used in combination with an AlexaFluor™488-labelled secondary donkey anti-rabbit antibody. Hundred microlitres of a 2% FBS solution were added to each well and the plate was kept on ice. The cell suspensions were analysed with a CytoFLEX instrument from Beckman Coulter.

To assess if MASP-1 can induce platelet activation, whole blood was supplemented with a contact pathway inhibitor (corn trypsin inhibitor, 30 µg/ml; Loxo GmbH) and acetylsalicylic acid (0.5 mg/ml; A5376, Merck). The samples were warmed at 37°C for 5 min. The blood was recalcified (final concentration 12.5 mM) before the addition of a recombinant active form of MASP-1 (26, 27) (rMASP-1cf) (10 µg/ml and 50 µg/ml) and fixed at different time points. To further evaluate the mechanism of platelet activation, the platelet activation experiments were repeated in the presence of 100 nM of the PAR4 inhibitor BMS 986120 (Caymanchem, USA) and/or hirudin (525 ATU/ml; Sarstedt, Germany).

## Results

We used a microvascular bleeding model to investigate the role of complement lectin pathway components in haemostasis upon vessel injury. The model consists of channels within a silicone chip that are coated with fibronectin and collagen followed by a viable confluent monolayer of human endothelial cells. During a perfusion experiment, fresh human whole blood is flown through the endothelialised channels. Upon infliction of a mechanical injury, the haemostatic process can be observed in real-time under the confocal microscope by staining for fibrin and (activated) platelets or other proteins or cells of interest.

### MBL accumulates at the injury site

**Figure 2:**
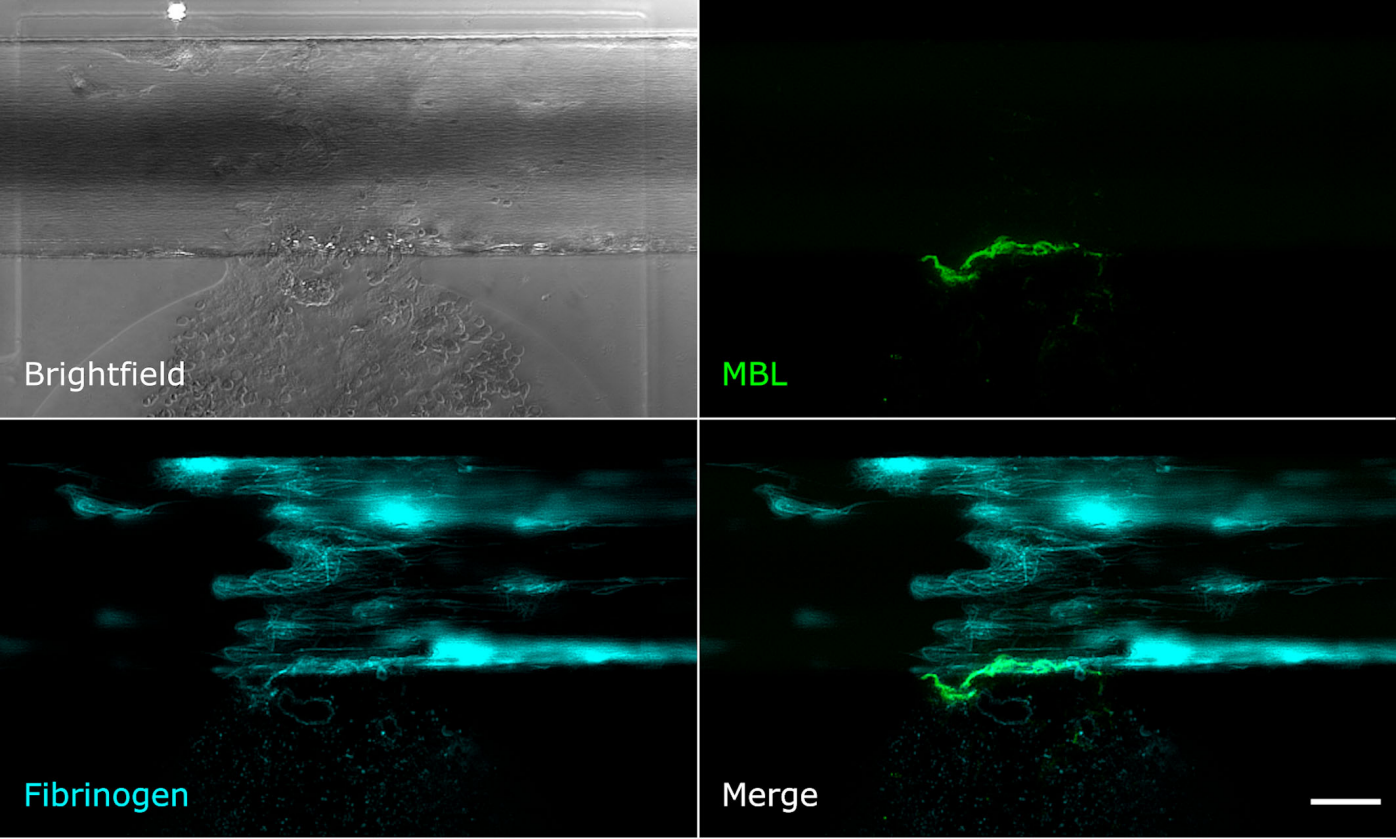
MBL and fibrin accumulate upon vessel injury. Microfluidic bleeding model 20 min after injury. From top left to bottom right: Brightfield, anti-MBL signal (green), AlexaFluor647-conjugated fibrin signal (cyan), and merged signals. MBL accumulates at the injury site in a wall-like structure but shows only partial colocalisation with fibrin. Scale bar: 50 µm.

Upon mechanical vessel injury, a bloodstream exiting the artificial vessel could be observed (**Figure 2**, brightfield image). Depending on the size of the injury, the blood was flowing out of the vessel for up to 40 min until the clot formed was strong enough to stop the bleeding. The fibrinogen/fibrin signal was already recognisable in the first image of the time series (1 min after injury) at the injury site and on the injured cells. The signal increased over time and fibrin filaments became recognisable (**Figure 2**). The MBL signal was also recognisable in the first image of the time series, but only at the edge of the vessel directly at the injury site. Over time, a wall-like structure became recognisable where the blood flowed out of the vessel (**Figure 2**). Next, we aimed to explore the structure/s or protein/s MBL was binding to.

### MBL shows only little colocalisation with fibrin, but strong colocalisation with platelets and von Willebrand factor

**Figure 3:**
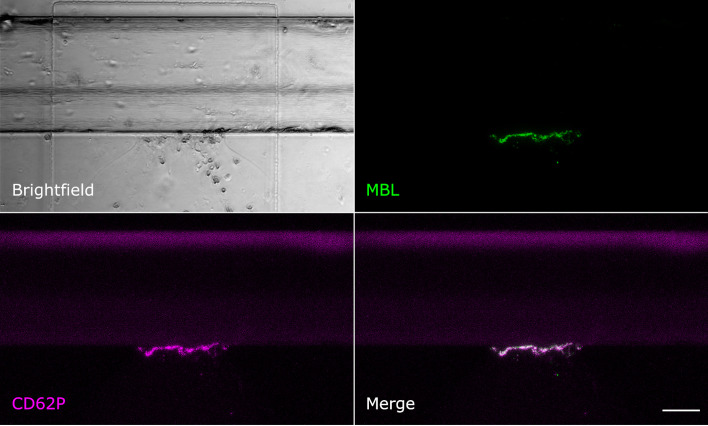
MBL colocalises with activated platelets. Microfluidic bleeding model 60 min after injury. From top left to bottom right: Brightfield, anti-MBL signal (green), anti-CD62P signal (purple), and merged signals. CD62P-expressing activated platelets and MBL showed a complete colocalisation. Scale bar: 50 µm.

**Figure 4:**
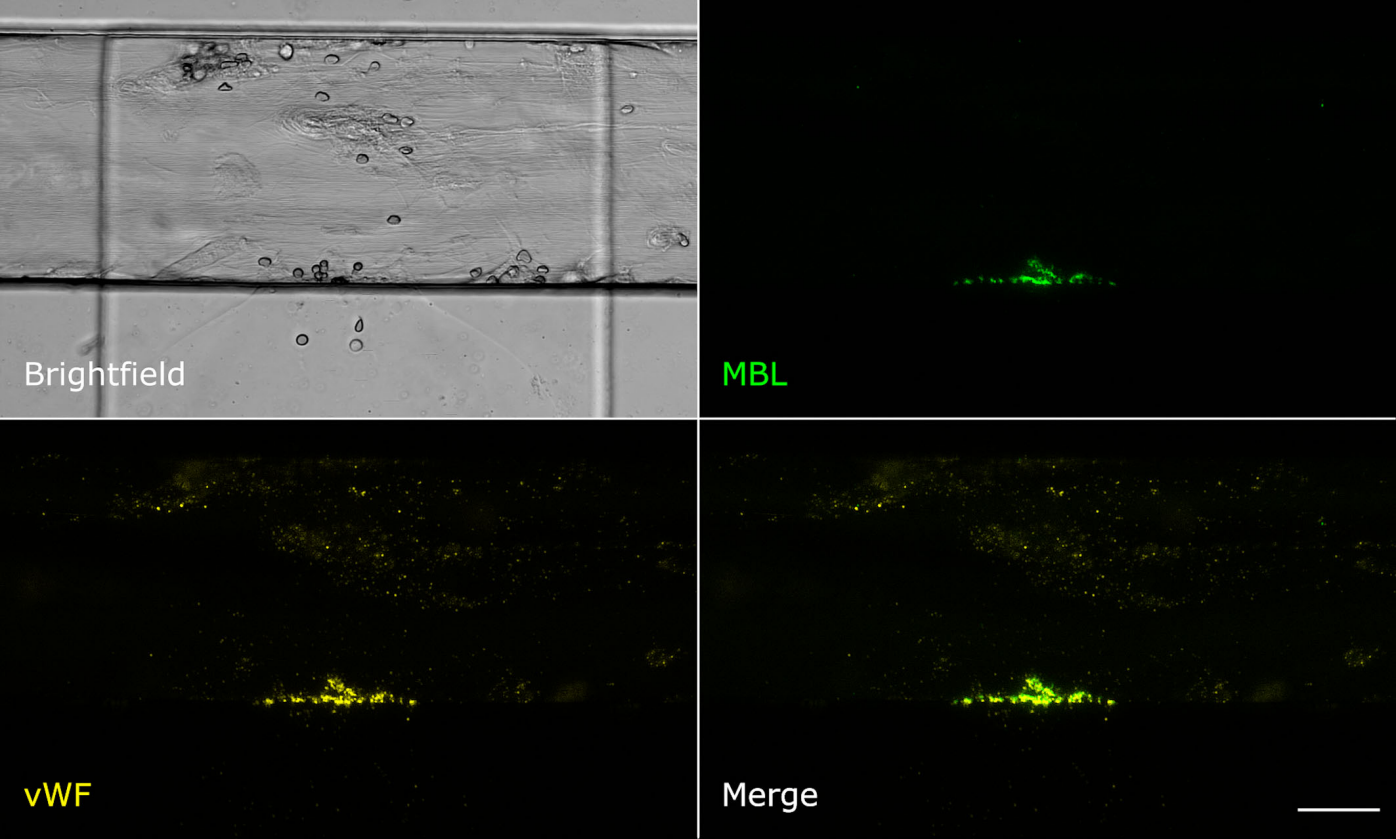
MBL colocalises with von Willebrand factor. Microfluidic bleeding model 45 min after injury. From top left to bottom right: Brightfield, anti-MBL signal (green), anti-von Willebrand factor signal (yellow), and merged signals. MBL and von Willebrand factor showed strong colocalisation. Scale bar: 50 µm.

Even though MBL and fibrin were both present at the injury site, their signals showed only little colocalisation. Fibrin was detected throughout the vessel around the injury site and also in the collagen pouch where it formed filaments. The MBL signal on the other hand was clearly limited to the injury site. This MBL-containing wall-like structure showed only a small extent of colocalisation with fibrin (**Figure 2**). We also tested for colocalisation with crosslinked fibrin, but again, only a small extent of colocalisation limited to the injury site was observed (**Supplementary Data 1**). Interestingly, experiments with an anti-CD41 platelet antibody (**Supplementary Data 1**) revealed a strong colocalisation with a similar wall-like structure, suggesting that MBL might be accumulating on platelets. However, the MBL signal was present to a smaller extent than the CD41 signal, suggesting that MBL may not bind to all platelets. We repeated the experiment with an anti-CD62P (P-selectin) antibody, a marker of activated platelets, and observed complete colocalisation of MBL and CD62P-expressing platelets (**Figure 3**). In addition, there was some colocalisation of MASP-1 and CD62P (**Supplementary Data 1**). We also detected von Willebrand factor from the first image of the time series and observed strong colocalisation of MBL with von Willebrand factor at the injury site (**Figure 4**).

### MBL shows no direct interactions with platelets in flow cytometry analyses

In order to verify if MBL could indeed bind to platelets and if MASP-1, which is in complex with MBL, could contribute to platelet activation, we performed flow cytometry analyses. Upon platelet activation with ADP in whole blood, the majority of the platelet population shifted from CD41-positive to CD62P-positive, however, neither non-activated nor activated platelets were MBL-positive (**Supplementary Data 2**).

### MASP-1 activates platelets by a thrombin-dependent mechanism

**Figure 5:**
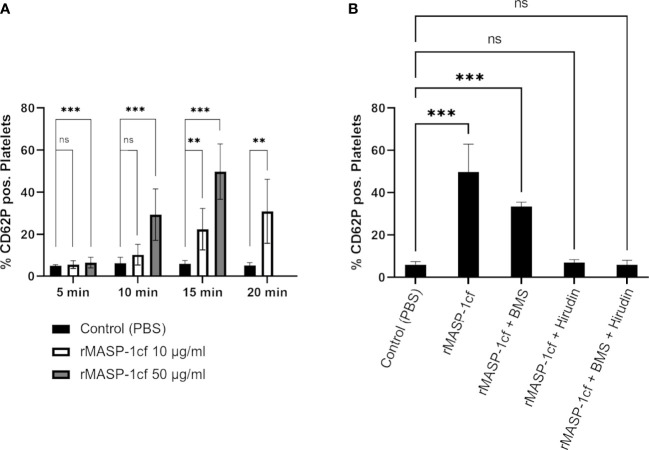
MASP-1 and platelet activation. Whole blood supplemented with corn trypsin inhibitor and acetylsalicylic acid was re-calcified and incubated with rMASP-1cf (10 μg/ml and 50 μg/ml) and fixed at different time points. The entire platelet population was identified with an anti-CD41 antibody conjugated with APC. The activated population was identified with an anti-CD62P antibody conjugated with BV421. **(A)** Addition of rMASP-1cf increased the percentage of platelets expressing CD62P (shown as mean with SD) in a time- and dose-dependent manner. Statistical analysis: The Shapiro-Wilk test confirmed normal distribution, and p-values for differences between groups were determined with an ANOVA test (ns not significant, * p ≤ 0.05, ** p ≤ 0.01, *** p ≤ 0.001). Number of experiments: Control: n=8; 10 μg/ml MASP-1: n=3; 50 μg/ml MASP-1: n=6). **(B)** The effect of 50 μg/ml rMASP-1cf was tested in the presence of PAR4 inhibitor BMS986120 and/or hirudin. The samples were fixed after 15min. Addition of the PAR4 inhibitor significantly reduced the effect of rMASP-1cf, while addition of hirudin canceled it out completely. Statistical analysis: The Shapiro-Wilk test confirmed normal distribution, and p-values for differences between groups were determined with an ANOVA test (ns not significant, * p ≤ 0.05, ** p ≤ 0.01, *** p ≤ 0.001). Number of experiments: Control: n=8; MASP-1 only: n=6; MASP-1+ BMS: n=3; MASP-1+ Hirudin: n=4; MASP-1+ BMS + Hirudin: n=4.

When we added recombinant active catalytic fragment of MASP-1 (rMASP-1cf) to whole blood and analysed platelet CD62P expression by flow cytometry, we observed a time- and dose-dependent increase in the number of activated platelets (**Figure 5A**).

Earlier studies have shown that MASP-1 is able to activate prothrombin (11, 12) and induce PAR4 receptor signalling on endothelial cells (28). In order to investigate whether MASP-1 could activate platelets through these mechanisms, we repeated the experiments with rMASP-1cf in the presence of the PAR4 inhibitor BMS 986120 and hirudin as a thrombin inhibitor. BMS 986120 is a selective PAR-4 receptor antagonist that had no enzyme inhibitory activity when tested against a panel of purified proteases, which included thrombin and other coagulation enzymes (29), making activity against MASP-1 highly unlikely. Hirudin was shown not to inhibit MASP-1 (30). As shown in **Figure 5B**, PAR4 inhibition decreased the number of platelets activated, while thrombin inhibition completely abolished the effect of rMASP-1cf. These results indicated that the observed effect of MASP-1 on CD62P expression on platelets was indeed mediated by thrombin.

### Inhibiting MBL binding reduces platelet activation upon vessel injury without affecting fibrin formation and bleeding time

We aimed to investigate if targeting MBL would affect fibrin formation, platelet activation, and ultimately bleeding time.

**Figure 6:**
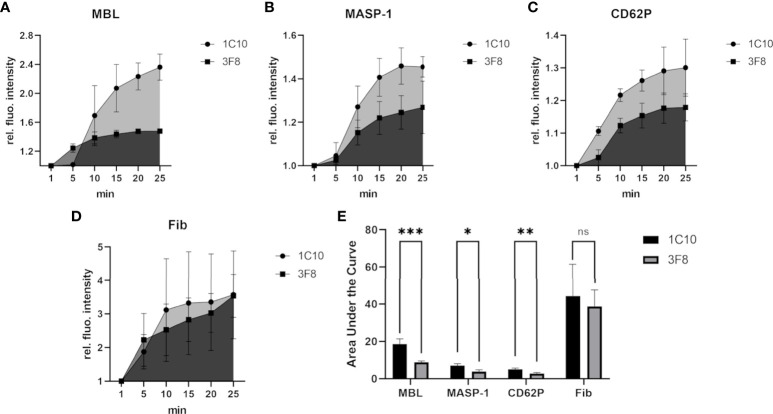
Effects of inhibiting MBL-binding in the bleeding model. To investigate the effects of the MBL inhibitory antibody 3F8, the signal intensity relative to the first time point was monitored for MBL **(A)**, MASP-1 **(B)**, activated platelets (CD62P) **(C)** and fibrin **(D)**. The area under the curve was calculated using GraphPad Prism **(E)**. Experiments using the inhibitory 3F8 antibody showed a reduced increase of the MBL, MASP-1 and CD62P signal in comparison to the non-inhibitory control antibody 1C10, but no clear difference in fibrin signals was detected. Each experiment was performed three times, data are shown as mean with SD, and p-values for differences between groups were determined with a t-test (ns not significant, * p ≤ 0.05, ** p ≤ 0.01, *** p ≤ 0.001).

The MBL inhibitory antibody 3F8 has been shown to bind within the carbohydrate recognition domain (CRD) of MBL and inhibit MBL-dependent complement activation, while the control antibody 1C10 binds to MBL without inhibitory effect (24, 31). As shown in **Figure 6**, the MBL inhibitory antibody 3F8 clearly reduced the incorporation of MBL (**Figure 6A**) and MASP-1 (**Figure 6B**) into the clot, and also reduced platelet activation (**Figure 6C**). On the other hand, fibrin deposition was not affected (**Figure 6D**). The observed effects of the MBL inhibitory antibody proved statistically significant (**Figure 6E**), however, they did not translate into differences in bleeding time (data not shown).

### Inhibiting MASP-1 significantly affects haemostasis upon vessel injury

**Figure 7:**
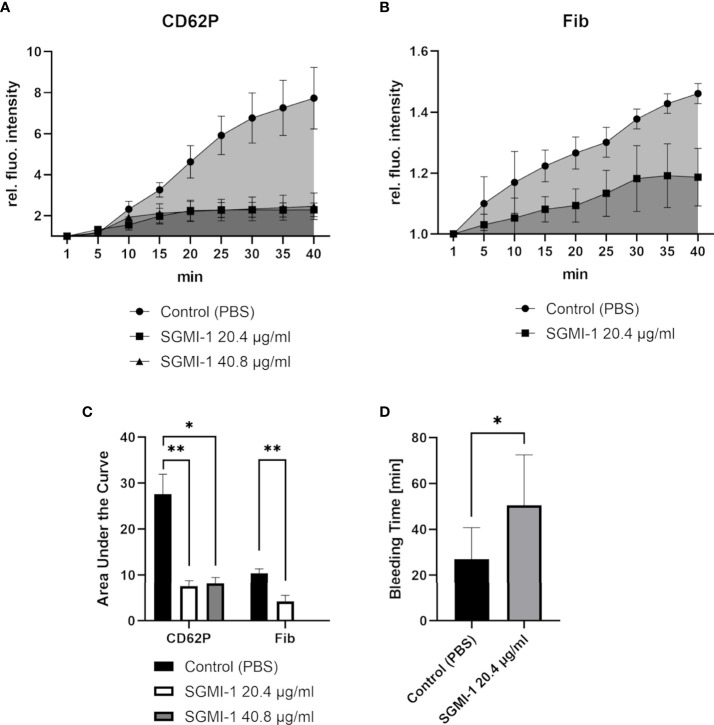
Effects of MASP-1-inhibition in the bleeding model. To investigate the effects of the MASP-1 inhibitor SGMI-1, the signal intensity relative to the first time point was monitored for activated platelets (CD62P) **(A)** and fibrin **(B)**. A) Both concentrations of SGMI (20.4 μg/ml; n=4 and 40.8ug/ml; n=2) resulted in a reduced increase of the CD62P signal in comparison to the control (n=6). B) Fibrin deposition was reduced in the presence of SGMI-1 (n=3). The area under the curve **(C)** was measured using GraphPad Prism. **(D)** SGMI-1 prolonged the bleeding time (n=3). Data are shown as mean with SD, and p-values for differences between groups were determined with ANOVA or t-test (* p ≤ 0.05, ** p ≤ 0.01, *** p ≤ 0.001).

Finally, we tested whether MASP-1 has significant effects in our haemostasis model. Inhibiting MASP-1 with the specific inhibitor SGMI-1 (25) reduced platelet activation measured as CD62P expression (**Figure 7A**) and fibrin formation (**Figure 7B**) to a statistically significant extent (**Figure 7C**) and prolonged bleeding time (**Figure 7D**). Compared with the control experiment without inhibitor, on average, MASP-1 inhibition nearly doubled the relative, injury size corrected bleeding time (**Figure 7D**).

## Discussion

Close interactions between the complement system and the coagulation cascade have been recognised. A better understanding of this interplay is important for the management of bleeding and thrombotic complications in a myriad of diseases ranging from trauma, infectious diseases and sepsis, autoimmune diseases, to diabetes, atherosclerosis and cardiovascular diseases.

The central components of the complement lectin pathway, MBL and its associated serine proteases, MASPs, have raised special interest in recent years due to the similarity and substrate overlap between MASPs and coagulation serine proteases. We and others have shown that MBL and MASPs interact with fibrin (15, 16), MASP-2 can activate coagulation factors and induce clot formation (10, 11), and MASP-1 can activate prothrombin, factor XIII and TAFI, enhance clot formation in a microfluidic model, and affect fibrinolysis (11–14, 27, 32). Lectin pathway components may therefore be a promising novel target in thrombosis (20, 21).

Haemostasis, on the contrary, is the physiological process to stop bleeding and prevent blood loss in the case of vessel injury. It seems likely that lectin pathway components would also support haemostasis, and first evidence has been reported from MBL and MASP-1 knock out mice exhibiting prolonged bleeding times *in vivo* (17). However, the underlying mechanisms, and whether lectin pathway components play a relevant role in haemostasis have not been clarified in a system that is close to the human *in vivo* situation. Here we used a microvascular bleeding model that allows to observe the haemostatic response in real-time in an injured artificial blood vessel lined with human endothelial cells and perfused with human whole blood.

We showed that MBL accumulated at the injury site immediately after the exposure of blood to injured cells and collagen. The observation of MBL forming a well-defined wall-like structure directly at the injury site is a novel and intriguing finding. Interestingly, the major binding site for MBL seemed not to be fibrin, which may seem in contrast to findings by Endo et al. (15). Note that in the study by Endo et al. (15), the experiments were performed with purified or recombinant mouse proteins or mouse serum, and human MBL differs from the mouse forms, emphasising the importance of validating animal results in human systems. Kozarcanin et al. (16) performed ELISA-based binding assays in plasma and found that neither MBL nor ficolins bound to fibrin, but MASP-1 and MASP-2 bound to purified fibrin D-dimer fragments. Fibrin D-dimer is generated through the crosslinking action of activated FXIII. We did detect some colocalisation of MBL with crosslinked fibrin at the injury site. Of course, colocalisation does not prove direct binding, MBL can bind to fibrin D-dimer indirectly through the associated MASP subunits or both MBL and fibrin can bind to the same underlying structure.

In our study, MBL colocalised at the injury site with von Willebrand factor (vWF). It was recently shown that components of the alternative complement pathway can assemble and activate on anchored ultra-large vWF multimeric strings (33). It was also shown that C3 can bind to the vWF A3 region (34). In our model, both vWF and MBL appeared in the same location immediately after vessel injury. This congruence in both time and space may indicate that MBL plays a role in the very first steps of haemostasis and raises the possibility that MBL directly binds to vWF.

We also found MBL to colocalise with platelets, and in particular with activated platelets, however, our flow cytometry analysis ruled out direct binding of MBL to platelets, which was in agreement with the results of Kozarcanin et al. (16). This suggests that MBL and platelets bind to the same structure, e.g. vWF. As a consequence, MBL-associated MASP-1, – which we also detected at the injury site of the bleeding model – could then contribute to platelet activation. Since MASP-1 can activate endothelial cells through the PAR4 receptor (28), it was plausible that platelets could also be activated by binding of MASP-1 to the platelet PAR4 receptor. Our flow cytometry results confirmed that MASP-1 can induce platelet activation, which is consistent with observations in a murine thrombosis model (21). However, our results obtained in the presence of PAR4- and thrombin inhibitors suggested that the platelet-activating effect of MASP-1 is mainly mediated by thrombin. Interestingly, Kozarcanin et al. (16) proposed that activated platelets and fibrin could in turn also activate MASP-1 and MASP-2, as assessed by complex-formations between these MASPs and the C1-inhibitor and antithrombin III serpins. Nevertheless, no direct evidence for MASP-1/2 activity was presented.

Finally, we explored whether targeting MBL or MASP-1 would affect the haemostatic response upon vessel injury. By blocking the carbohydrate-recognising domain of MBL with an antibody, both MBL-incorporation, and as expected, MBL-associated MASP-1 incorporation into the clot were reduced. Although platelet activation at the injury site was also reduced, fibrin formation and bleeding time were not affected. In sharp contrast, addition of the specific MASP-1 inhibitor SGMI-1 resulted in reduced platelet activation and fibrin formation at the injury site, and this also translated into prolongation of the bleeding time. The observation of a lacking effect of the MBL inhibitory antibody on fibrin deposition is in line with other observations. It emphasises again that MBL does not directly interact with fibrin. And it points again towards the crucial role of MASP-1 in promoting fibrin formation: when MASP-1 is inhibited directly by SGMI-1, a significant reduction of fibrin deposition could be observed (**Figure 7**). Using the MBL inhibitory antibody does probably not result in sufficient inhibition of MASP-1 activation to lead to a visible reduction in fibrin deposition.

Taken together, our results present the first evidence in a human *in vitro* model, that MBL and MASP-1 of the complement lectin pathway may support the haemostatic response and protect against bleeding upon mechanical vessel injury. The great advantage of our bleeding model is the possibility to observe reactions in real-time in the context of an endothelialised artificial vessel and whole blood under flow conditions. This also allows to study the sequence of events and their relevance in a complex environment. Most naturally, our first-in-a-row study has some limitations. In terms of scope, here we decidedly focused on MBL and MASP-1 as major lectin pathway initiators, but similar studies will be conducted to assess the roles of ficolins and MASP-2. This sharp focus was necessitated by the fact that working with freshly drawn whole blood obtained from anonymous healthy blood donors limited the number of experiments conductible with the same sample. We decided to make similar observations on a focused scope but in a large variety of donors to make our results more robust and more relevant to the real world compared to those results obtained by standardised purified samples or pooled normal plasma samples. Interestingly, despite suggestions that up to 40% of the population may have MBL deficiency, we always detected the MBL signal with any donor blood sample, supporting the view that observations of functional deficiency do not necessarily mean lack of MBL protein (35).

In conclusion, we show in a microvascular human bleeding model that MBL and in particular its associated enzyme MASP-1 have an important role in the haemostatic response triggered by mechanical vessel injury: the injury site is recognised by MBL and MASP-1 increases fibrin formation and platelet activation which in turn shortens the bleeding time. The lectin pathway may have evolved as one redundant system to support the crucial haemostatic system. While it may be beneficial during a physiological response, it may be harmful in the context of pathological thrombosis.

## Data availability statement

The raw data supporting the conclusions of this article will be made available by the authors, without undue reservation.

## Ethics statement

Ethical review and approval was not required for the study on human participants in accordance with the local legislation and institutional requirements. Written informed consent for participation was not required for this study in accordance with the national legislation and the institutional requirements.

## Author contributions

MG, JK and LJ performed the microfluidic bleeding model experiments. MG also performed the cytometry experiments and statistical analysis. EH and WL designed the microfluidic bleeding model and provided crucial materials for this study. JD, PG, GP and BK produced and provided recombinant MASP-1 (rMASP-1cf) and the MASP-1 inhibitor (SGMI-1) and contributed to data interpretation. VS designed and supervised the study. MG wrote the first draft of the manuscript. All authors contributed to manuscript revision, read, and approved the submitted version.

## Funding

This project was funded by grants awarded to VS by the Swiss National Science Foundation, grant number 310030_166413 (Bern, Switzerland), OPO Foundation (Zurich, Switzerland), Novartis Foundation for Medical-Biological Research (Basel, Switzerland), and Gottfried & Julia Bangerter-Rhyner Foundation (Bern, Switzerland). Other grant contributions to GP and PG were provided by project no. 2018-1.2.1-NKP-2018-00005 implemented with the support provided from the National Research, Development and Innovation Fund of Hungary, financed under the 2018-1.2.1-NKP funding scheme and by the National Research, Development and Innovation Office (Hungarian Scientific Research Fund) grants K119374, K119386, KH130376 and K135289.

## Acknowledgments

We would like to thank Prof. Peter Garred, University of Copenhagen, Denkmark, for kindly providing the MBL inhibitory antibody 3F8 and 1C10 control antibody. We would like to thank Prof. Kenneth Clemetson, University of Bern, Switzerland, for helpful discussions regarding the platelet experiments.

## Conflict of interest

The authors declare that the research was conducted in the absence of any commercial or financial relationships that could be construed as a potential conflict of interest.

## Publisher’s note

All claims expressed in this article are solely those of the authors and do not necessarily represent those of their affiliated organizations, or those of the publisher, the editors and the reviewers. Any product that may be evaluated in this article, or claim that may be made by its manufacturer, is not guaranteed or endorsed by the publisher.

## References

[1] Ricklin D Hajishengallis G Yang K Lambris JD. Complement: a key system for immune surveillance and homeostasis. Nat Immunol (2010) 11:785–97. doi: 10.1038/ni.1923 PMC2924908 20720586

[2] Dunkelberger JR Song W-C. Complement and its role in innate and adaptive immune responses. Cell Res (2010) 20:34–50. doi: 10.1038/cr.2009.139 20010915

[3] Rawlings ND Barrett AJ Bateman A. MEROPS: the peptidase database. Nucleic Acids Res (2010) 38:D227–33. doi: 10.1093/nar/gkp971 PMC2808883 19892822

[4] Rawlings ND. Peptidase inhibitors in the MEROPS database. Biochimie (2010) 92:1463–83. doi: 10.1016/j.biochi.2010.04.013 20430064

[5] Conway EM. Reincarnation of ancient links between coagulation and complement. J Thromb Haemost (2015) 13:S121–32. doi: 10.1111/jth.12950 26149013

[6] Oikonomopoulou K Ricklin D Ward PA Lambris JD. Interactions between coagulation and complement–their role in inflammation. Semin Immunopathol (2012) 34:151–65. doi: 10.1007/s00281-011-0280-x PMC3372068 21811895

[7] Mollnes TE Garred P Bergseth G. Effect of time, temperature and anticoagulants on *in vitro* complement activation: consequences for collection and preservation of samples to be examined for complement activation. Clin Exp Immunol (1988) 73:484–8. PMC1541764 2463123

[8] Keragala CB Draxler DF McQuilten ZK Medcalf RL. Haemostasis and innate immunity – a complementary relationship: A review of the intricate relationship between coagulation and complement pathways. Br J Haematol (2018) 180:782–98. doi: 10.1111/bjh.15062 29265338

[9] Huber-Lang M Sarma JV Zetoune FS Rittirsch D Neff TA McGuire SR. Generation of C5a in the absence of C3: a new complement activation pathway. Nat Med (2006) 12:682–7. doi: 10.1038/nm1419 16715088

[10] Krarup A Wallis R Presanis JS Gál P Sim RB. Simultaneous activation of complement and coagulation by MBL-associated serine protease 2. PloS One (2007) 2:e623. doi: 10.1371/journal.pone.0000623 17637839 PMC1910608

[11] Gulla KC Gupta K Krarup A Gal P Schwaeble WJ Sim RB. Activation of mannan-binding lectin-associated serine proteases leads to generation of a fibrin clot. Immunology (2010) 129:482–95. doi: 10.1111/j.1365-2567.2009.03200.x PMC2842495 20002787

[12] Hess K Ajjan R Phoenix F Dobó J Gál P Schroeder V. Effects of MASP-1 of the complement system on activation of coagulation factors and plasma clot formation. PloS One (2012) 7:e35690. doi: 10.1371/journal.pone.0035690 22536427 PMC3335018

[13] Jenny L Dobo J Gal P Schroeder V. MASP-1 of the complement system promotes clotting *via* prothrombin activation. Mol Immunol (2015) 65:398–405. doi: 10.1016/j.molimm.2015.02.014 25745807

[14] Jenny L Dobó J Gál P Schroeder V. MASP-1 induced clotting - the first model of prothrombin activation by MASP-1. PloS One (2015) 10:e0144633. doi: 10.1371/journal.pone.0144633 26645987 PMC4672900

[15] Endo Y Nakazawa N Iwaki D Takahashi M Matsushita M Fujita T. Interactions of ficolin and mannose-binding lectin with fibrinogen/fibrin augment the lectin complement pathway. J Innate Immun (2009) 2:33–42. doi: 10.1159/000227805 20375621

[16] Kozarcanin H Lood C Munthe-Fog L Sandholm K Hamad OA Bengtsson AA. The lectin complement pathway serine proteases (MASPs) represent a possible crossroad between the coagulation and complement systems in thromboinflammation. J Thromb Haemost (2016) 14:531–45. doi: 10.1111/jth.13208 26614707

[17] Takahashi K Chang W-C Takahashi M Pavlov V Ishida Y La Bonte L. Mannose-binding lectin and its associated proteases (MASPs) mediate coagulation and its deficiency is a risk factor in developing complications from infection, including disseminated intravascular coagulation. Immunobiology (2011) 216:96–102. doi: 10.1016/j.imbio.2010.02.005 20399528 PMC2912947

[18] Polley MJ Nachman RL. Human platelet activation by C3a and C3a des-arg. J Exp Med (1983) 158:603–15. doi: 10.1084/jem.158.2.603 PMC2187348 6604123

[19] Sims PJ Wiedmer T. The response of human platelets to activated components of the complement system. Immunol Today (1991) 12:338–42. doi: 10.1016/0167-5699(91)90012-I 1755945

[20] Cervera A Planas AM Justicia C Urra X Jensenius JC Torres F. Genetically-defined deficiency of mannose-binding lectin is associated with protection after experimental stroke in mice and outcome in human stroke. PloS One (2010) 5:e8433. doi: 10.1371/journal.pone.0008433 20140243 PMC2815773

[21] La Bonte LR Pavlov VI Tan YS Takahashi K Takahashi M Banda NK. Mannose-binding lectin-associated serine protease-1 is a significant contributor to coagulation in a murine model of occlusive thrombosis. J Immunol (2012) 188:885–91. doi: 10.4049/jimmunol.1102916 PMC3253146 22156595

[22] Sakurai Y Hardy ET Ahn B Tran R Fay ME Ciciliano JC. A microengineered vascularized bleeding model that integrates the principal components of hemostasis. Nat Commun (2018) 9:509. doi: 10.1038/s41467-018-02990-x 29410404 PMC5802762

[23] Hardy ET Sakurai Y Lam WA. Miniaturized vascularized bleeding model of hemostasis. Methods Mol Biol (2022) 2373:159–75. doi: 10.1007/978-1-0716-1693-2_10 34520012

[24] Collard CD Väkevä A Morrissey MA Agah A Rollins SA Reenstra WR. Complement activation after oxidative stress: Role of the lectin complement pathway. Am J Pathol (2000) 156:1549–56. doi: 10.1016/S0002-9440(10)65026-2 PMC1876913 10793066

[25] Héja D Harmat V Fodor K Wilmanns M Dobó J Kékesi KA. Monospecific inhibitors show that both mannan-binding lectin-associated serine protease-1 (MASP-1) and -2 are essential for lectin pathway activation and reveal structural plasticity of MASP-2. J Biol Chem (2012) 287:20290–300. doi: 10.1074/jbc.M112.354332 PMC3370211 22511776

[26] Dobó J Harmat V Beinrohr L Sebestyén E Závodszky P Gál P. MASP-1, a promiscuous complement protease: structure of its catalytic region reveals the basis of its broad specificity. J Immunol (2009) 183:1207–14. doi: 10.4049/jimmunol.0901141 19564340

[27] Jenny L Dobó J Gál P Pál G Lam WA Schroeder V. MASP-1 of the complement system enhances clot formation in a microvascular whole blood flow model. PloS One (2018) 13:e0191292. doi: 10.1371/journal.pone.0191292 29324883 PMC5764403

[28] Megyeri M Makó V Beinrohr L Doleschall Z Prohászka Z Cervenak L. Complement protease MASP-1 activates human endothelial cells: PAR4 activation is a link between complement and endothelial function. J Immunol (2009) 183:3409–16. doi: 10.4049/jimmunol.0900879 19667088

[29] Wong PC Seiffert D Bird JE Watson CA Bostwick JS Giancarli M. Blockade of protease-activated receptor- 4(PAR4) provides robust antithrombotic activity with low bleeding. Sci Transl Med (2017) 9:371. doi: 10.1126/scitranslmed.aaf5294 28053157

[30] Presanis JS Hajela K Ambrus G Gál P Sim RB. Differential substrate and inhibitor profiles for human MASP-1 and MASP-2. Mol Immunol (2004) 40:921–9. doi: 10.1016/j.molimm.2003.10.013 14725788

[31] Zhao H Wakamiya N Suzuki Y Hamonko MT Stahl GL. Identification of human mannose binding lectin (MBL) recognition sites for novel inhibitory antibodies. Hybrid Hybridomics (2002) 21:25–36. doi: 10.1089/15368590252917610 11991814

[32] Jenny L Noser D Larsen JB Dobó J Gál P Pál G. MASP-1 of the complement system alters fibrinolytic behaviour of blood clots. Mol Immunol (2019) 114:1–9. doi: 10.1016/j.molimm.2019.07.005 31325724

[33] Turner NA Moake J. Assembly and activation of alternative complement components on endothelial cell-anchored ultra-large von willebrand factor links complement and hemostasis-thrombosis. PloS One (2013) 8:e59372. doi: 10.1371/journal.pone.0059372 23555663 PMC3612042

[34] Nolasco JG Nolasco LH Da Q Cirlos S Ruggeri ZM Moake JL. Complement component C3 binds to the A3 domain of von willebrand factor. TH Open (2018) 2:e338-45. doi: 10.1055/s-0038-1672189 31080944 PMC6508891

[35] Garred P Larsen F Madsen HO Koch C. Mannose-binding lectin deficiency–revisited. Mol Immunol (2003) 40:73–84. doi: 10.1016/S0161-5890(03)00104-4 12914814

