# Supplementary material for: Complement lectin pathway components MBL and MASP-1 promote haemostasis upon vessel injury in a microvascular bleeding model
Source: Front Immunol. 2022 Aug 12;13:948190. doi: 10.3389/fimmu.2022.948190 (PMC9412763; doi:10.3389/fimmu.2022.948190)
Supplement: Supplementary file 1 [file DataSheet_1.pdf]

Supplementary Material

1 Supplementary Figure 1

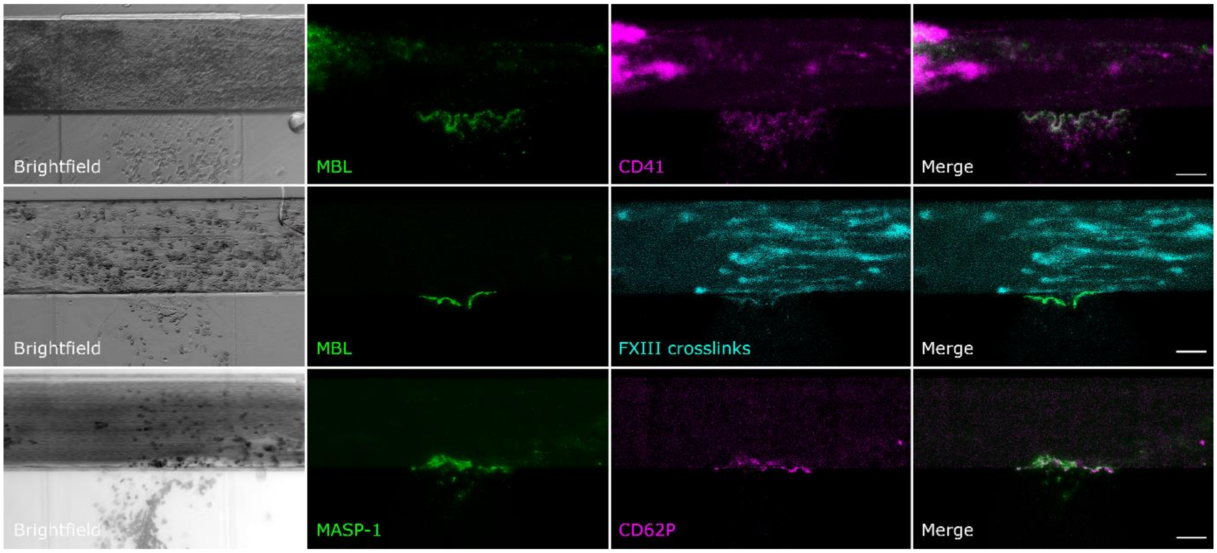

Colocalisation experiments between MBL or MASP-1 with CD41, FXIII-induced fibrin crosslinks, or CD62P, upon vessel injury in the microvascular bleeding model.

2 Supplementary Figure 2

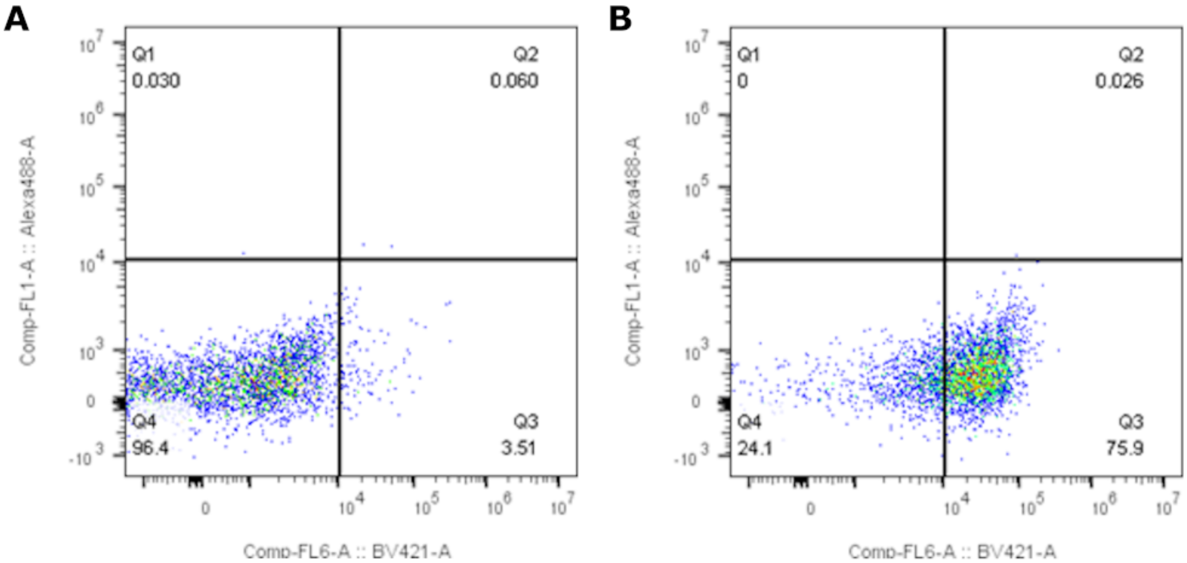

Platelet population (CD41-positive) before and after platelet activation. After recalcification of citrated whole blood samples, platelets were activated with ADP, fixed and subjected to flow cytometry analysis. The platelet population was selected with a CD41 antibody. On the X axis is the CD62P-BLV421 signal, which shows the number of activated platelets. The Y axis shows the Alexa488 signal, which would show the MBL-positive platelets. The cut-off was selected by measuring the signal of the secondary antibodies without the primary antibodies. Panel A shows the platelet population before activation with ADP. Panel B shows the platelet population after activation with ADP. Both platelet populations, before and after activation, were MBL-negative. (Representative figures from one experiment out of three experiments using three different blood donors.)
